# Successful General Anesthesia for Intertrochanteric Femur Fracture in a 101-Year-Old Patient: A Case Report

**DOI:** 10.7759/cureus.111901

**Published:** 2026-07-01

**Authors:** Elvin Kesimci, Denada Haka, Bahtiyar Haberal

**Affiliations:** 1 Department of Anesthesiology, Başkent University Faculty of Medicine, Ankara, TUR; 2 Department of Orthopedics and Traumatology, Başkent University Hospital, Ankara, TUR

**Keywords:** centenarian, general anesthesia, intertrochanteric fracture, perioperative management, super-elderly

## Abstract

Hip fractures in centenarian patients represent an extreme perioperative challenge, with very limited published data on anesthetic safety in this cohort. We report a 101-year-old woman with no significant comorbidities (American Society of Anesthesiologists (ASA) physical status III) who sustained an intertrochanteric femur fracture and underwent successful surgical fixation under general anesthesia. Hemodynamic stability was maintained throughout the procedure without the need for vasopressors or inotropic support. The postoperative course was entirely uneventful; the patient was mobilized with physiotherapy assistance on postoperative day 1 and discharged home on postoperative day 3 in good condition. This case demonstrates that, with thorough preoperative risk stratification and vigilant intraoperative monitoring, centenarian patients can safely undergo hip fracture surgery under general anesthesia. Reporting such cases is essential to expand the limited evidence base guiding perioperative management in the super-elderly population.

## Introduction

Hip fractures in centenarian patients represent an extreme perioperative challenge, with very limited published data on anesthetic safety in this cohort. We report a 101-year-old woman who sustained an intertrochanteric femur fracture and underwent successful surgical fixation under general anesthesia. She was classified as American Society of Anesthesiologists (ASA) physical status III due to the presence of well-controlled systemic comorbidities (hypertension and diabetes mellitus) in addition to advanced age. Hemodynamic stability was maintained throughout the procedure without the need for vasopressors or inotropic support. The postoperative course was entirely uneventful; the patient was mobilized with physiotherapy assistance on postoperative day 1 and discharged home on postoperative day 3 in good condition. This case demonstrates that, with thorough preoperative risk stratification and vigilant intraoperative monitoring, centenarian patients can safely undergo hip fracture surgery under general anesthesia. Reporting such cases is essential to expand the limited evidence base guiding perioperative management in the super-elderly population.

## Case presentation

Hip fractures are among the most common and consequential injuries in the elderly population, carrying significant morbidity, mortality, and socioeconomic burden. As global life expectancy continues to rise, clinicians are increasingly confronted with patients of extreme age, including centenarians - those aged 100 years or older. This demographic is particularly challenging from a perioperative standpoint, as age-related physiological decline affects cardiovascular reserve, pulmonary function, renal clearance, and drug pharmacokinetics [1].

Surgical fixation remains the standard of care for intertrochanteric femur fractures, even in the very elderly, as conservative management is associated with significantly worse outcomes, including higher mortality, pressure ulcers, and prolonged immobility [2]. However, the optimal anesthetic technique for this age group remains debated, and the literature specifically addressing centenarian patients is extremely limited. Most published series group patients aged 80 or 90 years and older together, obscuring the unique physiological and anesthetic challenges of the 100 plus cohort [3].

From an anesthetic perspective, the management of centenarian patients presents unique challenges due to marked age-related physiological changes, including reduced cardiovascular reserve, altered autonomic responsiveness, and increased sensitivity to anesthetic agents [1]. These factors increase the risk of perioperative hemodynamic instability regardless of the anesthetic technique used. Regional anesthesia is often favored in elderly patients undergoing hip fracture surgery due to its potential to reduce systemic drug exposure and postoperative cognitive dysfunction. While neuraxial techniques are often preferred in elderly patients due to their potential to reduce systemic drug exposure and attenuate stress responses, they are also associated with clinically relevant hypotension, which may be poorly tolerated in patients with limited cardiovascular reserve. Conversely, general anesthesia remains a valid alternative in selected patients. It allows for controlled ventilation and titratable anesthetic depth but may be associated with concerns regarding cognitive outcomes and systemic drug effects in the very elderly. However, evidence directly comparing anesthetic techniques specifically in centenarian patients is extremely limited, and most available data are extrapolated from younger elderly cohorts. thereby obscuring the unique physiological and clinical characteristics of centenarians. Thus, there remains a significant gap in the literature regarding optimal anesthetic management in this extreme age group.

We present a case of a 101-year-old female patient who underwent surgical fixation of an intertrochanteric femur fracture under general anesthesia, contributing to the limited body of evidence by describing the uneventful perioperative management of a centenarian patient with careful hemodynamic control and early postoperative recovery.

## Discussion

This case highlights the perioperative management of a centenarian patient undergoing surgical fixation of an intertrochanteric femur fracture under general anesthesia with an uneventful perioperative course. The super-elderly population (≥100 years) represents a rapidly growing but still underrepresented group in anesthetic literature, and evidence guiding optimal perioperative management in this cohort remains limited [1,2].

From a physiological perspective, extreme age is associated with marked reductions in cardiovascular reserve, impaired autonomic responsiveness, decreased pulmonary compliance, reduced renal and hepatic clearance, and increased sensitivity to anesthetic and sedative agents [3]. These changes increase susceptibility to perioperative hypotension, delayed drug elimination, and postoperative cognitive dysfunction regardless of anesthetic technique [3,4].

Published literature on centenarian surgical patients is sparse. Available case series suggest that perioperative complication rates are elevated in this cohort; however, carefully selected patients with a limited comorbidity burden can achieve outcomes comparable to those of younger elderly patients [3,5].

Hip fracture surgery is among the most common emergency procedures in the elderly, and anesthetic management remains a subject of ongoing debate. Several large observational studies and randomized controlled trials comparing general and neuraxial anesthesia in elderly patients undergoing hip fracture surgery have demonstrated no consistent difference in mortality, although neuraxial techniques may be associated with reduced thromboembolic risk and opioid consumption [5,6]. However, neuraxial anesthesia can result in significant sympathetic blockade and clinically relevant hypotension, which may be poorly tolerated in patients with limited cardiovascular reserve [6,7].

Conversely, general anesthesia allows controlled ventilation, airway protection, and titratable anesthetic depth. Concerns persist regarding postoperative delirium and potential systemic effects of anesthetic agents in elderly patients; however, recent evidence suggests that the choice of anesthetic technique may not be independently associated with delirium when perioperative management is optimized [8].

Age alone should not preclude surgical intervention or the use of general anesthesia. Each case demands individualized assessment, transparent risk-benefit discussion with the patient and family, and a coordinated multidisciplinary perioperative plan. Outcome data from such cases are essential to inform future clinical decision-making and the development of evidence-based guidelines for anesthetic management in the super-elderly population.

In the present case, the decision to proceed with general anesthesia was based on individualized assessment, including preserved functional status prior to injury and absence of significant comorbidities. A low-dose, titratable anesthetic technique with sevoflurane and remifentanil infusion was used to maintain hemodynamic stability without vasopressor support. This approach is consistent with strategies emphasizing opioid-sparing and hemodynamically controlled anesthesia in frail elderly patients [3,4].

Early mobilization, effective multimodal analgesia, and absence of postoperative delirium further support the feasibility of carefully managed general anesthesia in selected centenarian patients undergoing hip fracture surgery. However, due to the inherent limitations of a single case report, no causal inference or superiority of anesthetic technique can be established.

## Conclusions

In conclusion, this case describes the successful perioperative management of a centenarian patient undergoing surgical fixation of an intertrochanteric femur fracture under general anesthesia. The procedure was completed without major hemodynamic instability, vasopressor requirement, or perioperative complications in this specific case. While this observation suggests that general anesthesia may be feasible in carefully selected centenarian patients when guided by individualized perioperative planning, the findings are inherently limited by the nature of a single case report. Therefore, no generalizable conclusions regarding the superiority or safety of anesthetic techniques can be drawn. Further detailed case series and comparative studies are required to better define optimal anesthetic strategies in this growing and high-risk population.
